# Ancestry-attenuated effects of socioeconomic deprivation on type 2 diabetes disparities in the *All of Us* cohort

**DOI:** 10.1186/s44263-023-00025-2

**Published:** 2023-11-06

**Authors:** Vincent Lam, Shivam Sharma, Sonali Gupta, John L. Spouge, I. King Jordan, Leonardo Mariño-Ramírez

**Affiliations:** 1 National Institute on Minority Health and Health Disparities, National Institutes of Health, 11545 Rockville Pike, Building 11545 Rockville Pike, 2WF Room C14, Rockville, MD 20818, USA.; 2 School of Biological Sciences, Georgia Institute of Technology, Atlanta, GA, USA.; 3 National Library of Medicine, National Institutes of Health, Bethesda, MD, USA.

**Keywords:** Health disparities, Diabetes, Race, Ethnicity, Genetic ancestry, Socioeconomic deprivation, Interactions, *All of Us*

## Abstract

**Background:**

Diabetes is a common disease with a major burden on morbidity, mortality, and productivity. Type 2 diabetes (T2D) accounts for roughly 90% of all diabetes cases in the USA and has a greater observed prevalence among those who identify as Black or Hispanic.

**Methods:**

This study aimed to assess T2D racial and ethnic disparities using the *All of Us* Research Program data and to measure associations between genetic ancestry (GA), socioeconomic deprivation, and T2D. We used the All of Us Researcher Workbench to analyze T2D prevalence and model its associations with GA, individual-level (iSDI), and zip code-based (zSDI) socioeconomic deprivation indices among participant self-identified race and ethnicity (SIRE) groups.

**Results:**

The study cohort of 86,488 participants from the four largest SIRE groups in *All of Us*: Asian (*n* = 2311), Black (*n* = 16,282), Hispanic (*n* = 16,966), and White (*n* = 50,292). SIRE groups show characteristic genetic ancestry patterns, consistent with their diverse origins, together with a continuum of ancestry fractions within and between groups. The Black and Hispanic groups show the highest levels of socioeconomic deprivation, followed by the Asian and White groups. Black participants show the highest age- and sex-adjusted T2D prevalence (21.9%), followed by the Hispanic (19.9%), Asian (15.1%), and White (14.8%) groups. Minority SIRE groups and socioeconomic deprivation, both iSDI and zSDI, are positively associated with T2D, when the entire cohort is analyzed together. However, SIRE and GA both show negative interaction effects with iSDI and zSDI on T2D. Higher levels of iSDI and zSDI are negatively associated with T2D in the Black and Hispanic groups, and higher levels of iSDI and zSDI are negatively associated with T2D at high levels of African and Native American ancestry.

**Conclusions:**

Socioeconomic deprivation is associated with a higher prevalence of T2D in Black and Hispanic minority groups, compared to the majority White group. Nonetheless, socioeconomic deprivation is associated with reduced T2D risk within the Black and Hispanic groups. These results are paradoxical and have not been reported elsewhere, with possible explanations related to the nature of the *All of Us* data along with SIRE group differences in access to healthcare, diet, and lifestyle.

## Background

Diabetes is a pervasive and costly disease in the USA and beyond, affecting over 34.2 million Americans [1] and costing billions of dollars annually in both healthcare expenses and reduced productivity [2]. Of those afflicted with diabetes in the USA, type 2 diabetes (T2D) accounts for roughly 90% of cases [3]. T2D disproportionately affects minority racial and ethnic groups in the USA, with greater prevalence rates being observed among Hispanic, non-Hispanic Asian, and non-Hispanic Black individuals than among non-Hispanic White individuals [1, 4, 5]. In addition to minority race and ethnicity, it has been demonstrated that socioeconomic deprivation increases one’s risk of developing T2D [6, 7]. T2D risk has also been shown to vary with genetic ancestry, with European ancestry being associated with lower odds of developing T2D and African and Native American ancestry being associated with greater odds of developing T2D [8–11].

The causes of T2D are complex and span a multitude of genetic, demographic, social, and environmental factors that may modulate one another [12, 13]. As such, insight into the epidemiology of T2D may be gleaned from observing how these factors act in concert to affect disease risk. One promising source of data from which such insight may be obtained is the United States (US) National Institutes of Health (NIH) All of Us Research Program (abbreviated as *All of Us* hereafter) [14].

*All of Us* was launched in 2015 with the mission of advancing individualized healthcare and health equity through the collection of genetic and health data from thousands of individuals across the USA [14]. The program places a special emphasis on recruiting participants from populations that have been historically underrepresented in health research. The resulting diverse participant cohort provides rich opportunities to investigate how self-identified race, ethnicity, and genetic ancestry, as well as their respective interactions with socioenvironmental factors, influence T2D risk in the USA.

The first aim of this study was to determine whether there is evidence for T2D racial and ethnic health disparities in the *All of Us* cohort. The second aim was to assess whether and how SED, genetic ancestry, and their respective interactions contribute to T2D disparities and whether these contributions are consistent with what is known about how genetic and environmental factors influence T2D risk. We previously observed synergistic effects between socioeconomic deprivation and genetic ancestry in increasing T2D risk among ethnic minorities using data from the United Kingdom Biobank [15, 16]. The results of this study may reveal T2D disparity risk factors that could be potential targets of policy measures that aim to ameliorate the T2D disease burden in the USA. This study could also provide insight into the potential of *All of Us* as a resource for investigating epidemiological questions and health disparities.

## Methods

### Study cohort

The cohort for this study was assembled from participant data made available through the *All of Us* Researcher Workbench, a cloud-based platform through which registered researchers can access and analyze *All of Us* participant data. *All of Us* volunteer participants enroll directly through JoinAllofUs.org or at participating health care provider organizations. The participant cohort consists of adults aged 18 years and older who reside in the USA or in a US territory. Those who were either incarcerated or lacked the capacity to consent at the time of enrollment were excluded from the program. The *All of Us* operational protocol (#2016–05) was approved by the NIH Institutional Review Board.

*All of Us* participant data are available to registered researchers via the Researcher Workbench: https://www.researchallofus.org/data-tools/workbench/. Participant data consists of three datasets corresponding to different access tiers. The public tier dataset exclusively contains aggregate data and is freely accessible to all. The registered tier dataset consists of de-identified, individual-level data and is restricted to approved researchers. The controlled tier dataset consists of individual-level genomic data and expanded electronic health record (EHR) information. The study cohort was built using the *All of Us* Registered Tier Dataset v6 (curated version R2022Q2R2) and the *All of Us* Controlled Tier Dataset v6 (curated version C2022Q2R2). These datasets consist of data collected from participants who enrolled from 2018 to 2021, with a data cutoff date of January 1, 2022, and a data release date of June 22, 2022. Participant EHR, demographic, and socioeconomic data were obtained from the Registered Tier dataset. Participant genomic data were obtained from the Controlled Tier dataset. Demographic data consisted of self-identified race and ethnicity (SIRE), date of birth, and sex at birth. International Classification of Diseases codes (ICD-9-CM and ICD-10-CM) were extracted from participant EHR data and mapped to phecode 250.2 to classify individuals as T2D cases and controls [17]. Following the phecode convention, patients who exhibited phenotypes corresponding to phecodes 249–250.99 were excluded from the study cohort, as they bore conditions too similar to T2D to be reliably assigned as controls.

### Race and ethnicity

Following enrollment, *All of Us* participants responded to a number of surveys spanning topics concerning their background, lifestyle, and health. In a core survey titled “The Basics,” participants are asked to select one or more of seven main racial or ethnic categories that best describe them: (1) American Indian or Alaska Native, (2) Asian, (3) Black, (4) Hispanic or Latino, (5) Middle Eastern or North African, (6) Native Hawaiian or Pacific Islander, and (7) White. Additionally, participants could respond with “None of these fully describe me” or “Prefer not to answer.” The *All of Us* Researcher Workbench provides these data as self-identified race and ethnicity categories, following the US Office of Management and Budget Standards. This information is currently unavailable for individuals who self-identify as American Indian or Alaska Native. Our study cohort consists of four largest SIRE categories for *All of Us* participants: Asian, Black, Hispanic, and White. We defined non-Hispanic Asian, Black, and White participants as those who selected these respective racial categories in the “The Basics” core survey and no other racial or ethnic category. We defined Hispanic participants as all who selected “Hispanic or Latino.”

### Quantifying socioeconomic deprivation

To quantify socioeconomic deprivation, we used a zip code-based socioeconomic deprivation index (zSDI) devised by Brokamp et al. [18]. zSDI values are available for most participants in the *All of Us* Researcher workbench. Further motivating our decision to use zSDI as a metric for deprivation is the demonstrated role of geographic location in driving and perpetuating health disparities [19]. The zSDI is based on six different socioeconomic variables included in the 2015 American Community Survey [20]. The values of these variables are defined for census tracts and include (1) the proportion of the population whose income in the past 12 months placed them below the poverty threshold, (2) the median household income of the population, (3) the proportion of the population who are at least 25 years of age who have at least a high school level of education, (4) the proportion of the population with no health insurance coverage, (5) the proportion of households that received public assistance income or food stamps within the past 12 months, and (6) the proportion of households that are vacant. The zSDI represents the value of the first principal component resulting from a principal component analysis performed on these six measures. The zSDI ranges from 0 to 1, with a higher value corresponding to higher levels of socioeconomic deprivation.

*All of Us* Researcher Workbench survey questions related to socioeconomic status were used to create an individual-level socioeconomic deprivation index (iSDI) comparable to the area-level deprivation index created by Brokamp et al. [18]. The following questions from *All of Us*’s “The Basics” survey were used to calculate iSDI: (1) “What is your annual household income from all sources?”, (2) “Do you own or rent the place where you live?”, (3) “Are you covered by health insurance or some other kind of health care plan?”, (4) “What is your current employment status?”, and (5) “What is the highest grade or year of school you completed?”. We coded participant responses to these questions as ordinal variables and performed dimensionality reduction using principal component analysis of the ordinal values to compute iSDI for individual *All of Us* participants. Like the zSDI area-level deprivation index created by Brokamp et al. [18], this index ranges from 0 to 1, with greater values corresponding to higher levels of deprivation.

### Genetic ancestry

Genome-wide genotype data was made available for 165,080 participants through the *All of Us* Controlled Tier Dataset v6. Genotype variants were called for 1,824,517 genomic positions on the GRCh38/hg38 reference genome build using the Illumina Global Diversity Array. We harmonized *All of Us* participant genotype variants with whole genome sequence variant data from global reference populations characterized as part of the 1000 Genomes Project and the Human Genome Diversity Project [21, 22]. Biallelic variants that were shared across the *All of Us*, and the global reference population variant sets were merged, ensuring consistency between reference and alternate allele designations. Linkage disequilibrium (LD) pruning was performed with the parameters (1) window size = 50, (2) step size = 10, and (3) pairwise threshold r2 < 0.1. The final merged, harmonized, and LD-pruned genotype data set consists of 187,795 variants. Variant merging, harmonization, and LD were performed using PLINK version 1.9 [23].

The Rye (Rapid ancestrY Estimation) program was used to infer participant genetic ancestry from the final variant dataset [24]. Rye performs genetic ancestry inference by utilizing principal component analyses of genomic variant data. Principal component analysis was performed on the harmonized variant dataset using the FastPCA program implemented in PLINK version 2.0 [25, 26]. The resulting data was used to define reference samples representing six continental ancestry groups: African, Asian, European, Native American, Oceanian, and West Asian. Rye was run on the first 25 principal components of the data, assigning ancestry group fractions to each participant.

### Statistical analysis

Due to the overrepresentation of older and female participants in the study cohort, SIRE-specific T2D prevalence estimates were adjusted for age and sex. For every SIRE group, unadjusted T2D prevalence, p, was taken to be K cases over n total individuals belonging to the SIRE group. Age/sex adjustment was performed by weighing the unadjusted prevalence for groups of participants corresponding to different age-sex combinations using census fractions, f, calculated from American Community Survey 1-year estimates [20]. Census fractions, in this case, are the proportion of the total US population of a SIRE group that falls into a particular age-sex category. From different age-sex categories, c, adjusted prevalence, $\hat{p}$, was calculated like so:

$$\hat{p}=\sum_{c\in C}\frac{f_{c}}{n_{c}}K_{c}$$

95% confidence intervals for each adjusted SIRE-specific prevalence estimate were calculated by adding and subtracting the product of each adjusted estimate’s standard error, $\sigma (\overset{ˆ}{p})$, and 1.96 to and from the adjusted prevalence estimate:

$$\sigma (\overset{ˆ}{p})=\sqrt{\sum_{c\in C}\;\frac{f_{c}^{2}}{n_{c}}\frac{K_{c}}{n_{c}}\left(1-\frac{K_{c}}{n_{c}}\right)}$$

$$p\epsilon [\overset{ˆ}{p}-1.96\times \sigma (\overset{ˆ}{p}),\overset{ˆ}{p}+1.96\times \sigma (\overset{ˆ}{p})]$$

All T2D model analyses were performed in R version 4.2.2 using the *stats* package [27]. Multivariable logistic regression was used to investigate associations between risk factors such as SIRE, genetic ancestry, and SDI on T2D case status (case = 1, control = 0). Participant age and sex were included as covariates in all models. Regression models were constructed using the glm function in R. To assess the effects of geographical clustering on our results, multilevel models were constructed using the glmer function from the lme4 package in R [28]. Interaction effects were also deduced with the glm function in R. These effects were visualized using the plot_model function from the sjPlot R package [29]. Specifications for all models can be found in the titles for their corresponding tables.

## Results

The cohort for this study was selected from *All of Us* participants who have EHR data available and who fell outside the exclusion range for T2D, as defined by the phecode exclusion scheme (Additional file 1: Fig. S1). Individuals in the cohort were restricted to those whose survey responses designated them to one of four SIRE groups—Asian, Black, Hispanic, and White—which represent the four largest racial or ethnic categories in the USA. The study cohort was further restricted to individuals for whom genomic and socioeconomic data were available and whose sex at birth was either male or female. Our final cohort consisted of 86,488 individuals whose mean age was 54.3 and of whom 64.78% were female (Table 1).

**Fig. 1:**
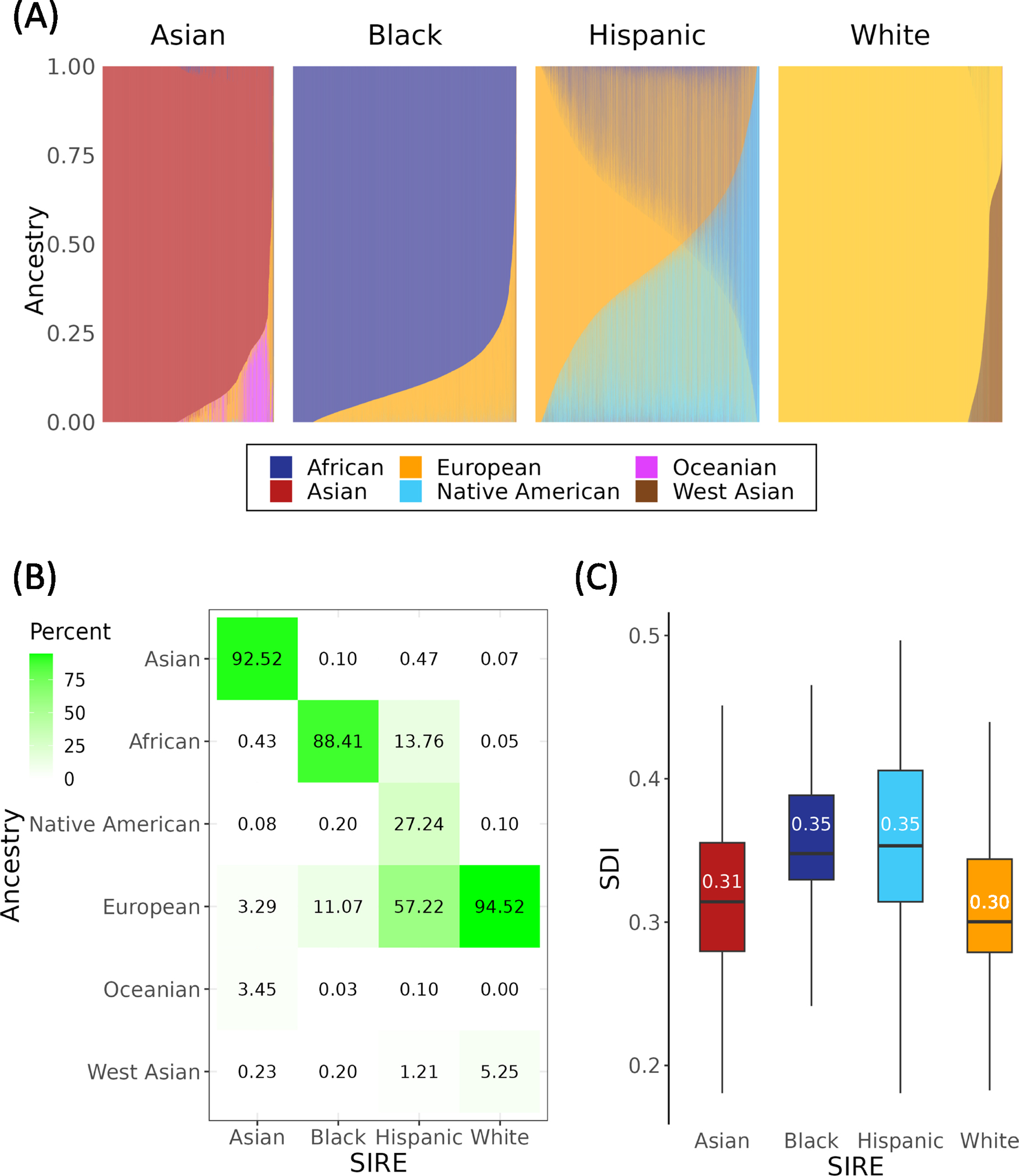
Genetic ancestry and socioeconomic deprivation in the *All of Us* cohort. **A** Genetic ancestry fractions are shown for six continental ancestry groups, stratified by participant self-identified race and ethnicity (SIRE). Stacked bar charts show the ancestry composition each individual, color-coded by ancestry as indicated in the key. **B** Average ancestry group fractions (*y*-axis) are shown for each SIRE group (*x*-axis). **C** Distributions of the socioeconomic deprivation index (zSDI) for each SIRE group. Box plots show the median and interquartile ranges, with median values indicated

*All of Us* participant genomic variant data were analyzed together with variant data from global reference populations to infer participants’ genetic ancestry fractions for six continental ancestry groups: African, Asian, European, Native American, Oceanian, and West Asian (Additional file 1: Table S1 and Fig. S2). Participant genetic ancestry fractions, stratified by SIRE groups, are shown in Fig. 1A. SIRE groups show characteristic ancestry patterns, together with continua of ancestry fractions within and between groups. Those who self-identified as belonging to the Asian SIRE group were predominantly of Asian ancestry, the Black SIRE group of African ancestry, and the White SIRE group of European ancestry (Fig. 1B). Some exceptions to this pattern can be noted however, as in the case of certain individuals belonging to the White SIRE group who were mostly of West Asian ancestry, and in the case of certain individuals belonging to the Black and Asian SIRE group who were mostly of European ancestry. In comparison to the Asian, Black, and White SIRE groups, individuals belonging to the Hispanic SIRE group demonstrated great heterogeneity in their ancestry fractions, with substantial European, Native American, and African components. This is consistent with the fact that participants with Hispanic ethnicity can identify with any race, following the current OMB standards.

*All of Us* participant socioeconomic deprivation was measured using a composite, place-based index (zSDI) that includes information on income, education, housing, and public assistance. We observe a clear disparity in zSDI across the four SIRE groups, with those self-identifying as Black and Hispanic exhibiting the highest median zSDI (0.35), followed by the Asian (0.31), and White (0.30) groups (Fig. 1C; ANOVA, *F* = 4793, *p* < 2e −16).

**Fig. 2:**
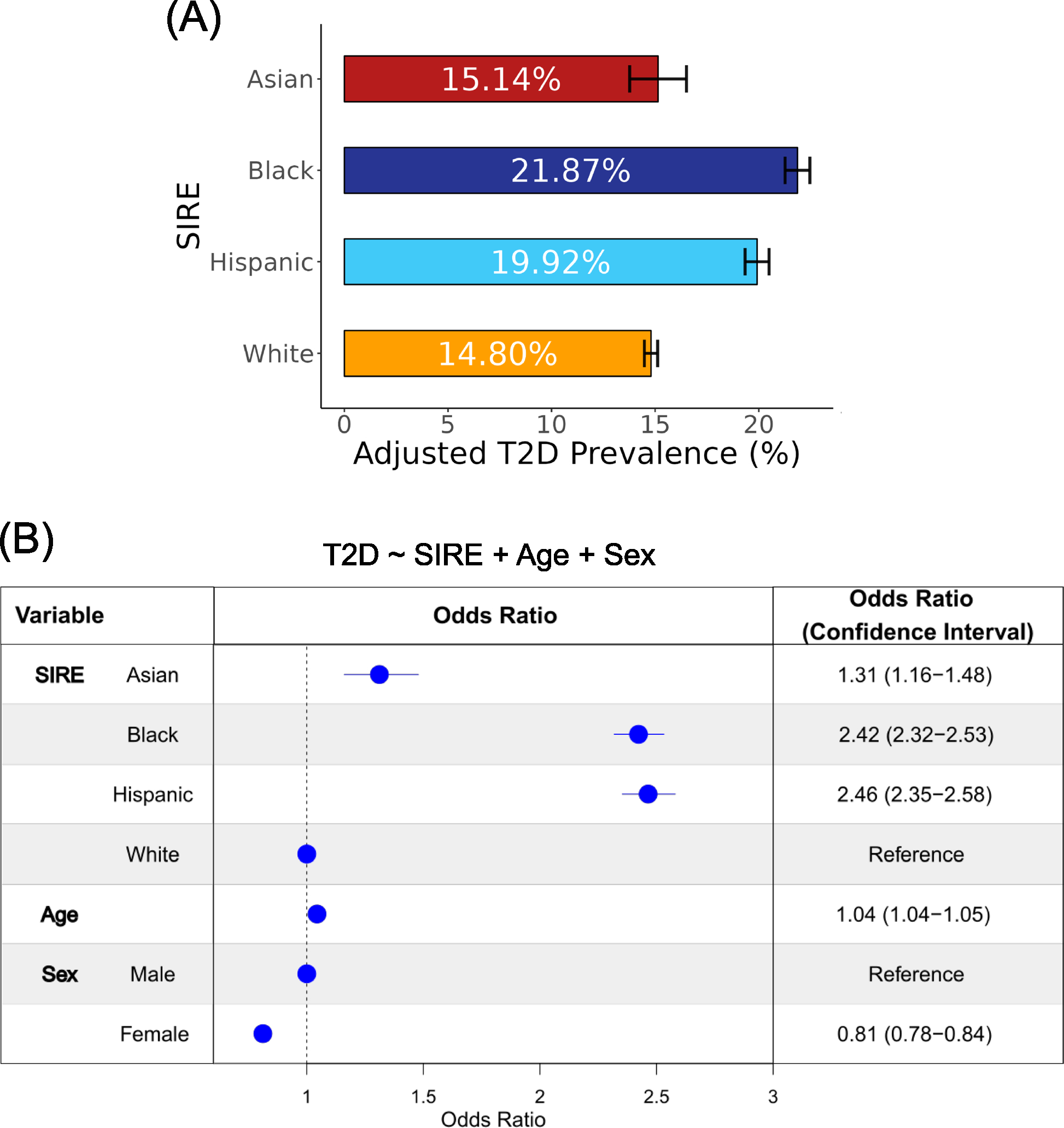
Type 2 diabetes (T2D) prevalence differences in the *All of Us* cohort. **A** Age and sex-adjusted average prevalence values, with 95% confidence intervals, are shown for participant self-identified race and ethnicity (SIRE) groups. **B** Odds ratios, with 95% confidence intervals, are shown for the multivariable logistic regression model: T2D ~ SIRE + age + sex. The odds ratio for age represents the change in T2D risk for every 1-year increase in age

Participant T2D diagnoses gleaned from EHR were used to calculate prevalence values for each of the four SIRE groups (Fig. 2A). Of these four groups, the Black SIRE group demonstrated the highest adjusted prevalence percentage (21.87%, CI 0.60) with the Hispanic SIRE group following closely behind (19.92%, CI 0.58). The Asian (15.14%, CI 1.37) and White (14.80%, CI 0.32) SIRE groups exhibit the lowest adjusted prevalence percentages. The relative T2D prevalence values among SIRE groups are similar to what is seen when different methods are used to create the cohort from *All of Us* data and resemble the pattern of T2D disparities reported by the Centers for Disease Control and Prevention (CDC; Additional file 1: Table S2).

To further investigate the association between T2D risk and SIRE, we modeled T2D case/control status as a function of SIRE, using age and sex as covariates (Fig. 2B). In this model, the White SIRE group was used as a reference group. The results of this model revealed that belonging to the Hispanic as opposed to belonging to the White SIRE group conferred the greatest increase in the odds of T2D (OR 2.46, CI 2.35–2.58), followed by belonging to the Black SIRE group (OR 2.42, CI 2.32–2.53) and followed last by belonging to the Asian SIRE group (OR 1.31, CI 1.16–1.48). Additionally, increasing age (OR 1.04, CI 1.04–1.05) is associated with greater predicted T2D risk and being female (OR 0.81, CI 0.78–0.84) is associated with lower predicted T2D risk.

A similar analysis was performed to elucidate the association between T2D risk and genetic ancestry. In this analysis, we modeled T2D case/control status as a function of a particular genetic ancestry fraction, using age and sex as covariates (Table 2). A model with these specifications was generated for four genetic ancestry groups that our four SIRE groups of interest are closely associated with: Asian ancestry (Asian SIRE), African (Black SIRE), European (White SIRE), and Native American (Hispanic SIRE). African ancestry has the highest positive coefficient (0.21), suggesting that there is an increased level of T2D risk among those with greater African ancestry fractions. Native American ancestry has the second-highest coefficient (0.14). Asian (−0.10) and European (−0.19) have negative coefficients, suggesting that there is lower T2D risk among those with a greater proportion of these ancestry fractions. All of these coefficients are significant at an alpha of 0.05. These patterns largely remained and were amplified when SDI was controlled for (Additional file 1: Table S3).

Additional models were created to investigate the association between T2D risk and socioeconomic deprivation, which modeled T2D case/control status as a function of area-based zSDI and individual-level iSDI, using age and sex as covariates. Participant area-based (zSDI) and individual-level (iSDI) socioeconomic deprivation are signifantly correlated (*r* = 0.26, *p* = < 2.2e −16). As would be expected, the model returned a high, positive coefficient for zSDI (2.52) and iSDI (1.99), indicating greater odds of T2D at greater levels of both area-based and individual-level socioeconomic deprivation. Multilevel modeling with iSDI as a fixed effect and zSDI as the random effect returned a high, positive coefficient for iSDI (1.98), suggesting that individual-level socioeconomic deprivation remains tightly associated with T2D risk when controlling for zip code clustering (Table 2).

**Fig. 3:**
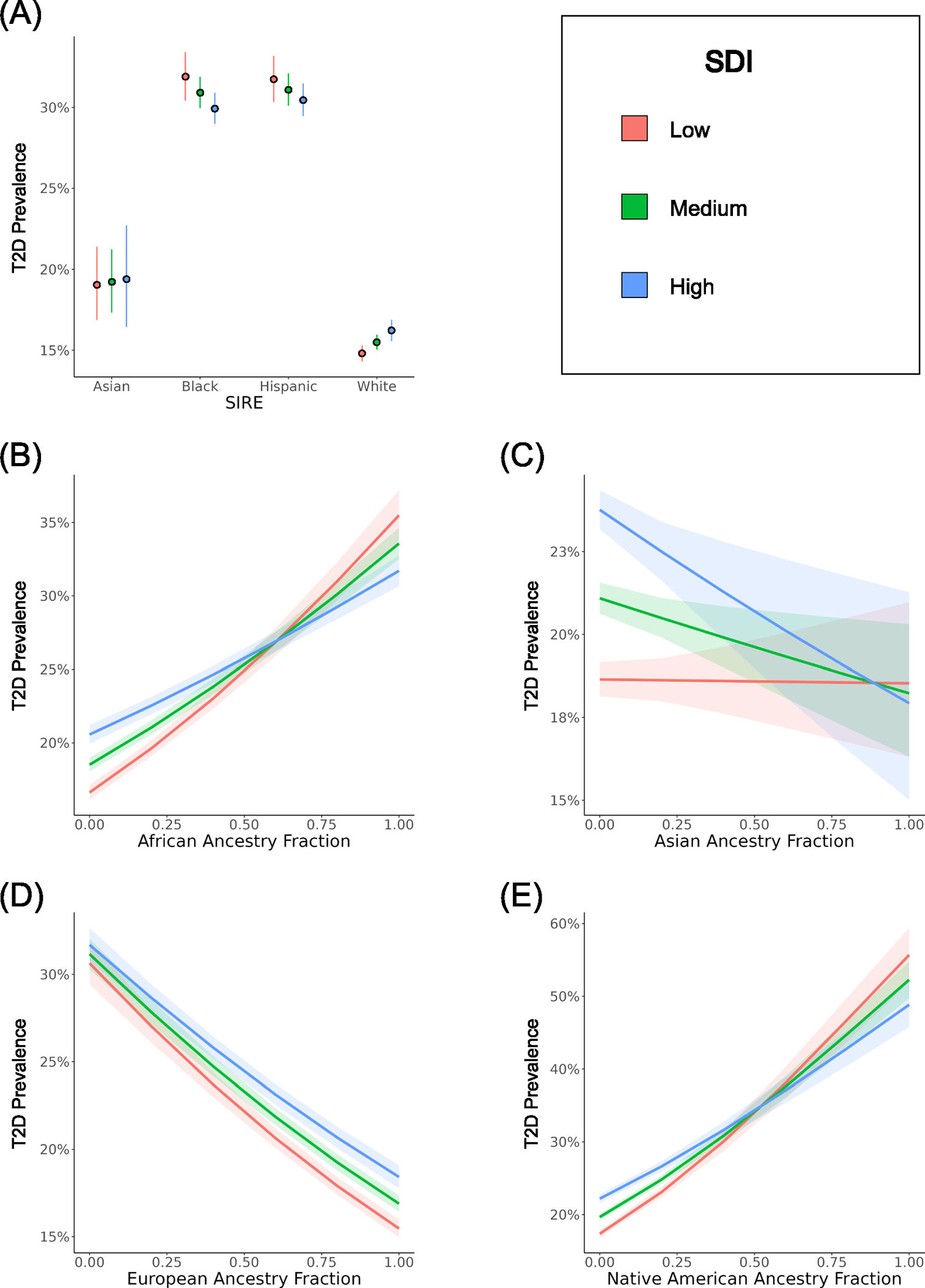
Interaction effects between race/ethnicity, genetic ancestry, and socioeconomic deprivation (zSDI) on T2D prevalence. T2D prevalence estimates, and 95% confidence intervals, are taken from multivariable logistic regression models that include interaction terms and are stratified by low (red), medium (green), and high (blue) zSDI. **A** Results for SIRE groups are based on the model T2D ~ SIRE*zSDI + age + sex. **B**–**E** Results for genetic ancestry (GA) fractions are based on the model T2D ~ GA*zSDI + age + sex

Additional multivariable logistic regression models were created to investigate how SIRE, genetic ancestry, and SDI interact to modify predicted T2D risk. As part of this analysis, we modeled T2D case/control status as a function of either the SIRE-zSDI or GA-zSDI interaction terms, using age and sex as covariates. The SIRE-zSDI models returned significant and negative interaction coefficients for the Black-zSDI (−1.67) and Hispanic-zSDI (−1.40) interaction terms, suggesting that greater socioeconomic deprivation is associated with reduced risk of T2D for individuals belonging to either the Black or Hispanic SIRE groups (Table 3 and Fig. 3A). However, when restricting the cohort to native-born participants, the Hispanic-zSDI interaction term is no longer significant (Additional file 1: Table S4). Relative excess of risk interaction (RERI) values for the Black (−4.02) and Hispanic (−3.66) groups are also negative, indicating subadditive effects of SIRE and zSDI. The opposite trend is observed for individuals belonging to the Asian and White SIRE groups, in which greater socioeconomic deprivation is associated with a greater risk of T2D. The negative interactions observed between Black and Hispanic SIRE and socioeconomic deprivation can also be seen when individuial-level iSDI is used to model T2D outcomes (Additional file 1: Table S5).

Similarly significant and negative interaction coefficients were returned by the GA-zSDI models, specifically for the African-zSDI (−3.59), Asian-zSDI (−2.90), and Native American-zSDI (−4.84) interaction terms (Table 3). In contrast, a significant positive interaction coefficient was reported for the European-zSDI (1.34) interaction term. RERI values show the same trends, with negative values for African-zSDI (−7.76), Asian-zSDI (−6.23), and Native American-zSDI (−10.48), compared to positive RERI for European-zSDI (2.18). Visualization of the GA-zSDI interactions shows that high zSDI is a risk factor for T2D at low levels of non-European ancestry and this trend switches at high levels of non-European ancestry, where low zSDI has a higher predicted risk (Fig. 3B–E). This pattern is particularly pronounced for African and Native American ancestry. The pattern of all of these interaction effects remains when the cohort is stratified by sex (Additional file 1: Table S6, Table S7, Fig. S3, and Fig. S4).

These results were validated with another set of models which modeled T2D risk as a function of zSDI, using age and sex as covariates (Table 4). These models were each run on a different subset of the study cohort consisting exclusively of individuals from one of the four SIRE groups under investigation. The models corresponding to the Black and Hispanic SIRE cohorts returned negative coefficients for zSDI (−0.70, −0.41). The model corresponding to the White SIRE cohort returned a significant and positive coefficient for zSDI (0.77), consistent with the coefficients observed for the SIRE-zSDI interaction terms.

## Discussion

The patterns observed in the SIRE group-specific T2D prevalence estimates align closely with patterns observed in other calculations of T2D prevalence (Additional file 1: Table S1). Across the different methods we used to calculate prevalence estimates using *All of Us* data, T2D prevalence was consistently highest among individuals identifying as Black and second highest among individuals identifying as Hispanic. Across almost all the methods we have used to calculate T2D prevalence, the prevalence was lowest among individuals identifying as White and second highest among individuals identifying as Asian. These disparities are consistent with group-specific T2D prevalence estimates shown in the CDC National Diabetes Statistics Report, supporting the use of *All of Us* to study T2D disparities [30].

This study validates previous investigations into the association between factors such as genetic ancestry, SIRE, and T2D risk. Past observations have shown that populations that are more socioeconomically deprived and consist of more individuals identifying as Black or Hispanic (or African or Native American ancestry) suffer from a greater T2D disease burden than their White and European ancestry counterparts [7–11]. The analyses that we performed on *All of Us* data reveal similar associations, underscoring the need for policy measures to alleviate the T2D disease burden of minority and socioeconomically deprived communities. What has been less thoroughly documented, however, is how interactions between genetic ancestry and socioeconomic deprivation are associated with T2D prevalence.

We previously explored interactions between socioeconomic deprivation and genetic ancestry using data obtained from the UK Biobank, a large-scale biomedical research resource that consolidates genetic and health data on roughly 500,000 participants from across the UK [15, 16]. These analyses revealed positive interaction effects between socioeconomic deprivation and ancestry, in contrast to the interaction effects reported here for the *All of Us* cohort, which returned significant negative interaction coefficients for all GA-zSDI interaction terms except European-zSDI. Negative interaction coefficients were also returned for all SIRE-zSDI interaction terms in this study. The negative Hispanic-zSDI term, however, is no longer significant when interaction analyses are run on a subset of the study cohort that exclusively consists of those native to the USA. This may be a result of the healthy immigrant paradox—a phenomenon in which immigrants have positive health outcomes relative to their socioeconomic status. This phenomenon was first reported among Hispanic immigrants living in the Southwestern USA [31]. The negative interactions between SIRE and zSDI are confirmed by zSDI effect size differences with and between groups. When the entire cohort is modeled together, zSDI is positively associated with T2D (Table 2), but zSDI is negatively associated with T2D for Black and Hispanic groups (Table 4).

That T2D risk may decrease with greater socioeconomic deprivation within minority groups contradicts prevailing knowledge concerning socioeconomic status and disease burden [32–35]. Barriers to healthcare access faced by members of minority groups may provide one possible explanation for these paradoxical results. Minority racial groups in the USA tend to face greater obstacles when pursuing medical care, as such groups have lower levels of health insurance coverage and reduced quality of care, among other challenges [36]. Presumably, these barriers are exacerbated by socioeconomic deprivation, so it may be the case that the most socioeconomically deprived members of minority groups are less likely to receive the level of medical care needed to receive a T2D diagnosis. Since our analysis relies on T2D diagnoses recorded in EHR, this could lower the observed T2D prevalence for the most socioeconomically deprived members of minority groups. If this conjecture is accurate, it would suggest that interventions are needed to expand healthcare access and encourage healthcare participation in socioeconomically deprived minority communities.

One additional explanation for the paradoxical trends observed here may be that the amounts of discrimination that racial minority groups are exposed to are greater at higher levels of socioeconomic status [37]. A previous study assessed the interaction effects between race, education, and education on the release of C-reactive protein (CRP), a biomarker of inflammation and general stress. The results of the study revealed that more highly educated individuals who identify as Black had elevated levels of CRP compared to their less-educated counterparts. Such differences were not as pronounced among individuals who identify as White. These findings suggest that due to higher levels of discrimination among those of greater socioeconomic status, better socioeconomic status may not strongly benefit the health of racial minority groups.

It could also be possible that, paradoxically, socioeconomic deprivation is a protective factor against T2D within minority racial groups. We have previously observed this phenomenon for an African ancestry population in Colombia, where those facing the most extreme forms of poverty have subsistence diet and lifestyle factors that contribute to a lower risk of T2D [11]. Similar occurrences may be contributing to lower T2D prevalence among the most deprived in the *All of Us* cohort. Those on the extreme ends of socioeconomic deprivation may also suffer from the most severe forms of food insecurity, which could contribute to a lower risk of T2D. Furthermore, as many social programs such as Medicaid and SNAP impose income cutoffs, the most deprived individuals may have access to medical and nutritional resources that moderately deprived individuals lack. A definitive resolution to this paradox will require further investigation.

There are several potential limitations to this study. As this is an observational study, a number of unobserved confounding variables may be present. Factors concerning lifestyle and diet, which are known to influence T2D risk, for instance, may covary with the three variables under investigation—SDI, genetic ancestry, and SIRE. Furthermore, population biobanks are prone to volunteer bias, whereby older, healthier, and less disadvantaged individuals tend to participate. This has been seen for the UK Biobank, but the extent of volunteer bias of *All of Us* is currently unknown [38]. Finally, the definition of T2D case status used here relies on diagnosis codes from EHR, which could lead to imprecise phenotyping, with potential differences across SIRE groups and levels of socioeconomic deprivation.

## Conclusions

In conclusion, these results provide evidence for the existence of T2D disparities in the *All of Us* participant cohort. While the T2D burden differs broadly across racial, genetic, and socioeconomic lines in ways previously reported, interactions between these variables reveal an unexpected interplay between genetic ancestry and socioeconomic status. The paradoxical relationship between socioeconomic status and T2D risk observed in Black and Hispanic individuals, though potentially a result of limited data, may reflect broader societal issues such as discrimination and inadequate access to healthcare among racial and ethnic minority groups. Policy interventions and research aimed at reducing racial health disparities may benefit from taking such issues into account.

## Supplementary Material

Supplementary Information **Additional file 1: Table S1.** Global reference populations used for genetic ancestry inference. **Table S2.** zSDI Interactions with race/ethnicity and genetic ancestry. **Table S3.** T2D genetic ancestry and SDI. **Table S4.** zSDI interactions with race/ethnicity and genetic ancestry among native-born participants. **Table S5.** iSDI interactions with race/ethnicity and genetic ancestry. **Table S6.** zSDI interactions with race/ethnicity and genetic ancestry among male participants. **Table S7.** zSDI interactions with race/ethnicity and genetic ancestry among female participants. **Fig. S1.** Flowchart for All of Us T2D cohort creation. **Fig. S2.** Principal component analysis of global reference populations and All of Us participants. **Fig. S3.** Male-stratified interaction effects between race/ethnicity, genetic ancestry, and socioeconomic deprivation (zSDI) on T2D prevalence. **Fig. S4.** Female-stratified interaction effects between race/ethnicity, genetic ancestry, and socioeconomic deprivation (zSDI) on T2D prevalence.

## Figures and Tables

**Table 1:** Study cohort

Characteristic	Full cohort (*n* = 86,488) (100%)	Asian (*n* = 2311) (2.67%)	Black (*n* = 16,282) (18.83%)	Hispanic (*n* = 16,966) (19.62%)	White (*n* = 50,929) (58.89%)

Mean age—years (SD)	55.31 (16.78)	47.70 (16.41)	52.93 (14.70)	48.20 (15.99)	58.78 (16.69)
Female (%)	56,030 (64.78)	1471 (63.65)	10,224 (62.79)	12,155 (71.64)	32,180 (63.19)
Male (%)	30,458 (35.22)	840 (36.35)	6,058 (37.21)	4811 (28.36)	18,749 (36.81)

**Table 2:** T2D, genetic ancestry, and SDI

Coefficient	Estimate	Standard error	*Z* value	*P* value

African	0.21	0.02	10.58	< 2e - 16
Asian	−0.10	0.05	−2.11	0.0348
European	−0.19	0.02	−9.81	< 2e - 16
Native American	0.14	0.05	−4.18	2.86e - 05
zSDI	2.52	0.14	17.59	< 2e - 16
ISDI-s	1.99	0.04	54.16	< 2e - 16
ISDI-m	1.98	0.04	49.29	< 2e - 16

T2D was modeled by ancestry, zipcode-based zSDI, and individual-level iSDI, controlling for age and sex. iSDI was modeled using single-level (iSDI-s) and multi-level (iSDI-m) models with iSDI-m as a fixed effect and zipcode as the random effect

**Table 3:** zSDI interactions with race/ethnicity and genetic ancestry

Coefficient	Estimate	Standard error	*Z* value	*P* value

**SIRE**				
Asian-zSDI	−0.71	1.07	−0.67	0.50
Black-zSDI	−1.67	0.39	−4.26	2.06e - 05
Hispanic-zSDI	−1.40	0.37	−3.80	1.43e - 04
**Genetic ancestry**				
Africa n-zS DI	−3.59	0.40	−8.91	< 2e - 16
Asian-zSDI	−2.90	1.11	−2.62	8.84e - 03
Europea n-zS DI	1.34	0.38	3.51	4.42e - 04
Native American-	−4.84	0.84	−5.77	8.18e - 09
zSDI				

T2D ~ SIRE*zSDI + age + sex, T2D ~ ancestry*zSDI + age + sex

**Table 4:** zSDI for SIRE-specific cohorts

Coefficient	Estimate	Standard error	*Z* value	*P* value

Asian	0.83	1.09	0.76	0.45
Black	−0.70	0.32	−2.19	0.03
Hispanic	−0.41	0.30	−1.39	0.16
White	0.77	0.23	3.34	8.38e - 04

T2D ~ zSDI + age + sex

## Ready to submit your research ? Choose BMC and benefit from:

- fast, convenient online submission
- thorough peer review by experienced researchers in your field
- rapid publication on acceptance
- support for research data, including large and complex data types
- gold Open Access which fosters wider collaboration and increased citations
- maximum visibility for your research: over 100M website views per year

## Abbreviations

CDC: Centers for Disease Control and Prevention
EHR: Electronic health record
GA: Genetic ancestry
LD: Linkage disequilibrium
NIH: National Institutes of Health
iSDI: Individual-level socioeconomic deprivation index
zSDI: Zip code-based socioeconomic deprivation index
SIRE: Self-identified race and ethnicity
T2D: Type 2 diabetes
US: United States
