# Supplementary material for: Ancestry-attenuated effects of socioeconomic deprivation on type 2 diabetes disparities in the All of Us cohort
Source: BMC Glob Public Health. Author manuscript; Available in PMC 2023 Dec 2. (PMC10693462; doi:10.1186/s44263-023-00025-2)
Supplement: Supplementary Information — Additional file 1: Table S1. Global reference populations used for genetic ancestry inference. Table S2. zSDI Interactions with race/ethnicity and genetic ancestry. Table S3. T2D genetic ancestry and SDI. Table S4. zSDI interactions with race/ethnicity and genetic ancestry among native-born participants. Table S5. iSDI interactions with race/ethnicity and genetic ancestry. Table S6. zSDI interactions with race/ethnicity and genetic ancestry among male participants. Table S7. zSDI interactions with race/ethnicity and genetic ancestry among female participants. Fig. S1. Flowchart for All of Us T2D cohort creation. Fig. S2. Principal component analysis of global reference populations and All of Us participants. Fig. S3. Male-stratified interaction effects between race/ethnicity, genetic ancestry, and socioeconomic deprivation (zSDI) on T2D prevalence. Fig. S4. Female-stratified interaction effects between race/ethnicity, genetic ancestry, and socioeconomic deprivation (zSDI) on T2D prevalence. [file NIHMS1944888-supplement-Supplementary_Information.docx]

**Ancestry-attenuated effects of socioeconomic deprivation on type 2 diabetes disparities**

Vincent Lam, Shivam Sharma, Sonali Gupta, John L. Spouge, I. King Jordan, Leonardo Mariño-Ramírez

**Contents**

[Table S1. **Global reference populations used for genetic ancestry inference.** 2](#_Toc146124941)

[Table S2. **zSDI Interactions with race / ethnicity and genetic ancestry.** 3](#_Toc146124942)

[Table S3. **T2D genetic ancestry and SDI.** 4](#_Toc146124943)

[Table S4. z**SDI interactions with race / ethnicity and genetic ancestry among native-born participants.** 5](#_Toc146124944)

[Table S5. i**SDI interactions with race / ethnicity and genetic ancestry.** 6](#_Toc146124945)

[Table S6. z**SDI interactions with race / ethnicity and genetic ancestry among male participants.** 7](#_Toc146124946)

[Table S7. **zSDI interactions with race / ethnicity and genetic ancestry among female participants.** 8](#_Toc146124947)

[Fig. S1. **Flowchart for *All of Us* T2D cohort creation.** 9](#_Toc146124948)

[Fig. S2. **Principal component analysis of global reference populations and *All of Us* participants.** 10](#_Toc146124949)

[Fig. S3. **Male-stratified interaction effects between race/ethnicity, genetic ancestry, and socioeconomic deprivation (zSDI) on T2D prevalence.** 11](#_Toc146124950)

[Fig. S4. **Female-stratified interaction effects between race/ethnicity, genetic ancestry, and socioeconomic deprivation (zSDI) on T2D prevalence.** 12](#_Toc146124951)

# Table S1. **Global reference populations used for genetic ancestry inference.**

| **Population** | **Source^a^** | **Number of samples** | **Continental Ancestry Group** |
| --- | --- | --- | --- |
| Balochi | HGDP | 17 | South Asian |
| Bedouin | HGDP | 15 | West Asian |
| Bougainville | HGDP | 9 | Oceanian |
| Brahui | HGDP | 20 | South Asian |
| CHB | 1KGP | 102 | East Asian |
| Colombian | HGDP | 6 | American |
| Dai | HGDP | 9 | East Asian |
| Druze | HGDP | 1 | West Asian |
| ESN | 1KGP | 99 | African |
| FIN | 1KGP | 85 | European |
| GBR | 1KGP | 87 | European |
| GIH | 1KGP | 2 | South Asian |
| GWD | 1KGP | 106 | African |
| IBS | 1KGP | 86 | European |
| ITU | 1KGP | 73 | South Asian |
| JPT | 1KGP | 104 | East Asian |
| Karitiana | HGDP | 12 | American |
| KHV | 1KGP | 96 | East Asian |
| LWK | 1KGP | 97 | African |
| Makrani | HGDP | 12 | South Asian |
| Maya | HGDP | 13 | American |
| MSL | 1KGP | 85 | African |
| Palestinian | HGDP | 41 | West Asian |
| Papuan Highlands | HGDP | 9 | Oceanian |
| Papuan Sepik | HGDP | 7 | Oceanian |
| PEL | 1KGP | 14 | American |
| Pima | HGDP | 12 | American |
| She | HGDP | 10 | East Asian |
| STU | 1KGP | 89 | South Asian |
| Surui | HGDP | 8 | American |
| TSI | 1KGP | 100 | European |
| Tujia | HGDP | 9 | East Asian |
| Tuscan | HGDP | 8 | European |
| YRI | 1KGP | 107 | African |

^a^ 1KGP – 1000 Genomes Project, HGDP – Human Genome Diversity Project

| Table S2. zSDI Interactions with race / ethnicity and genetic ancestry. “Phecode Cohort” refers to the cohort used for this study. In the “Double Rule Cohort”, only participants with at least two separate T2D diagnoses in their EHR are classified as cases. The “Survey Cohort” is comprised of individuals who have responded to an *All of Us* survey on endocrine diseases and assigns a case status to those who answered that they have been previously diagnosed with T2D. The “Cohort Builder Cohort” is comprised of all individuals who have EHR data present on the *All of Us* platform and who are in the four racial groups and two sexes of interest. Case status is assigned to all those with at least one T2D diagnosis in their EHR data. The last row features statistics taken from Appendix table 3 of Appendix A from the CDC National Diabetes Statistics Report and represent prevalence for types of diabetes from 2018-2019. | | | | |
| --- | --- | --- | --- | --- |
| Source | **Asian** | **Black** | **Hispanic** | **White** |
| Phecode Cohort | 15.14 (13.77-16.51) | 21.87 (21.27-22.47) | 19.92 (19.34-20.50) | 14.80 (14.48-15.12) |
| Double Rule Cohort | 13.09 (11.78-14.40) | 17.46 (16.91-18.01) | 16.46 (15.92-17.00) | 12.17 (11.88-12.46) |
| Survey Cohort | 7.26 (5.95-8.57) | 14.43 (13.29-15.57) | 10.71 (9.81-11.61) | 7.26 (6.99-7.53) |
| Cohort Builder Cohort | 3.68 (2.98-4.38) | 7.79 (7.41-8.17) | 6.42 (6.06-6.78) | 4.56 (4.39-4.73) |
| CDC National Diabetes Statistics Report | 9.5 (8.2–10.9) | 12.1 (11.3–13.0) | 11.8 (10.8–12.8) | 7.4 (7.1–7.7) |

| Table S3. T2D genetic ancestry and SDI. Adjusted Ancestry and SDI Models (Age, sex, and SDI are covariates) T2D ~ Ancestry + SDI + Age + Sex, T2D ~ iSDI + Age + Sex. The T2D ~ Ancestry analyses corresponding to Table 2 were re-run controlling for both the iSDIs we computed for each participant and zSDI. Additionally, we modeled T2D risk as a function of iSDI. These analyses were run to assess the contribution of individual and area-level deprivation to the influence of ancestry on T2D risk. It appears that the coefficients for each ancestry group increased in magnitude with this adjustment. iSDI, however, is not as strongly associated with T2D risk as Brokamp et al.’s area-based zSDI. | | | | |
| --- | --- | --- | --- | --- |
| T2D ~ Ancestry + iSDI + Age + Sex | | | | |
| Coefficient | **Estimate** | **Standard Error** | **Z value** | **P value** |
| African | 0.33 | 0.03 | 13.12 | < 2e-16 |
| Asian | 0.16 | 0.07 | 2.38 | 1.74e-02 |
| European | -0.47 | 0.02 | -19.34 | < 2e-16 |
| Native American | 0.95 | 0.05 | 17.58 | < 2e-16 |
| T2D ~ Ancestry + zSDI + Age + Sex | | | | |
| African | 0.73 | 0.02 | 29.89 | < 2e-16 |
| Asian | -0.14 | 0.07 | -2.08 | 3.79e-02 |
| European | -0.79 | 0.02 | -33.84 | < 2e-16 |
| Native American | 1.43 | 0.05 | 27.29 | < 2e-16 |

| Table S4. zSDI interactions with race / ethnicity and genetic ancestry among native-born participants. The analyses corresponding to Table 3 were re-run with a subset of our study cohort that exclusively consisted of individuals who were born in the United States. This was done to assess the potential impact that the healthy immigrant paradox may have on the results of our interaction models. | | | | |
| --- | --- | --- | --- | --- |
| Coefficient | **Estimate** | **Standard Error** | **Z value** | **P value** |
| SIRE | | | | |
| Asian-zSDI | -2.04 | 2.02 | -1.01 | 3.11e-01 |
| Black-zSDI | -1.87 | 0.40 | -4.63 | 3.70e-06 |
| Hispanic-zSDI | -0.63 | 0.51 | -1.23 | 2.20e-01 |
| Genetic ancestry | | | | |
| African-zSDI | -3.17 | 0.44 | -7.25 | 4.15e-13 |
| Asian-zSDI | -4.69 | 2.12 | -2.21 | 2.70e-02 |
| European-zSDI | 1.26 | 0.43 | 2.94 | 3.29e-03 |
| Native American-zSDI | -5.80 | 1.50 | -3.88 | 1.06e-04 |

| Table S5. iSDI interactions with race / ethnicity and genetic ancestry. T2D ~ SIRE*iSDI + age + sex, T2D ~ Ancestry*iSDI + age + sex | | | | |
| --- | --- | --- | --- | --- |
| Coefficient | **Estimate** | **Standard Error** | **Z value** | **P value** |
| SIRE | | | | |
| Asian-iSDI | -0.78 | 0.31 | -2.55 | 1.08e-02 |
| Black-iSDI | -1.82 | 0.10 | -18.26 | < 2e-16 |
| Hispanic-iSDI | -0.42 | 0.11 | -4.01 | 6.20e-05 |
| Genetic ancestry | | | | |
| African-iSDI | -2.34 | 0.10 | -22.27 | < 2e-16 |
| Asian-iSDI | -0.83 | 0.32 | -2.58 | 9.96e-03 |
| European-iSDI | 1.29 | 0.10 | 13.63 | < 2e-16 |
| Native American-iSDI | -0.94 | 0.25 | -3.72 | 1.97e-04 |

Table S6. z**SDI interactions with race / ethnicity and genetic ancestry among male participants.**

| Coefficient | Estimate | Standard Error | Z value | P value |
| --- | --- | --- | --- | --- |
| SIRE | | | | |
| Asian-zSDI | -1.20 | 1.64 | -0.73 | 4.63e-01 |
| Black-zSDI | -2.15 | 0.64 | -3.35 | 8.10e-04 |
| Hispanic-zSDI | -1.16 | 0.63 | -1.84 | 6.60e-02 |
| Genetic ancestry | | | | |
| African-zSDI | -3.40 | 0.67 | -5.03 | 4.81e-07 |
| Asian-zSDI | -2.37 | 1.70 | -1.40 | 1.63e-01 |
| European-zSDI | 1.77 | 0.61 | 2.91 | 3.56e-03 |
| Native American-zSDI | -2.29 | 1.49 | -1.53 | 1.26e-01 |

Table S7. **zSDI interactions with race / ethnicity and genetic ancestry among female participants.**

| Coefficient | Estimate | Standard Error | Z value | P value |
| --- | --- | --- | --- | --- |
| SIRE | | | | |
| Asian-zSDI | -0.39 | 1.41 | -0.27 | 7.83e-01 |
| Black-zSDI | -1.51 | 0.50 | -3.00 | 2.72e-03 |
| Hispanic-zSDI | -1.68 | 0.47 | -3.60 | 3.21e-04 |
| Genetic ancestry | | | | |
| African-zSDI | -3.90 | 0.51 | -7.72 | 1.19e-14 |
| Asian-zSDI | -3.01 | 1.47 | -2.04 | 4.09e-02 |
| European-zSDI | 1.34 | 0.49 | 2.70 | 6.95e-03 |
| Native American-zSDI | -6.34 | 1.02 | -6.20 | 5.78e-10 |


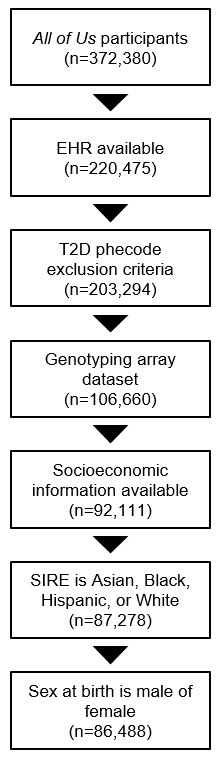


Fig. S1. **Flowchart for *All of Us* T2D cohort creation.** The inclusion/exclusion criteria, along with the number of participants retained, are shown for each step in the cohort creation.


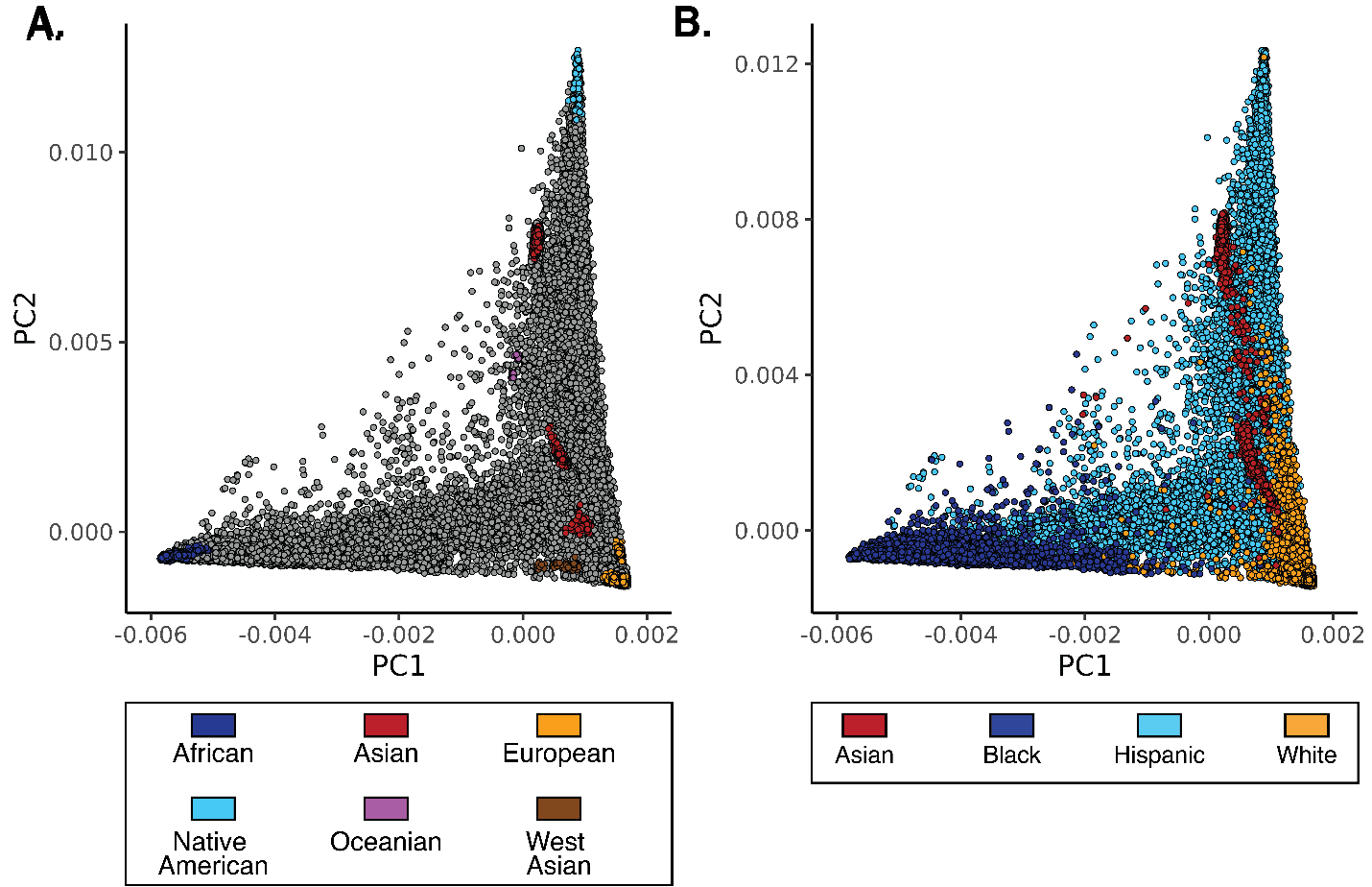


Fig. S2. **Principal component analysis of global reference populations and *All of Us* participants.** (A) Global reference populations are color-coded as shown in the key, and *All of Us* participants are shaded in gray. (B) All of Us participants are color-coded according to their self-identified race and ethnicity (SIRE).


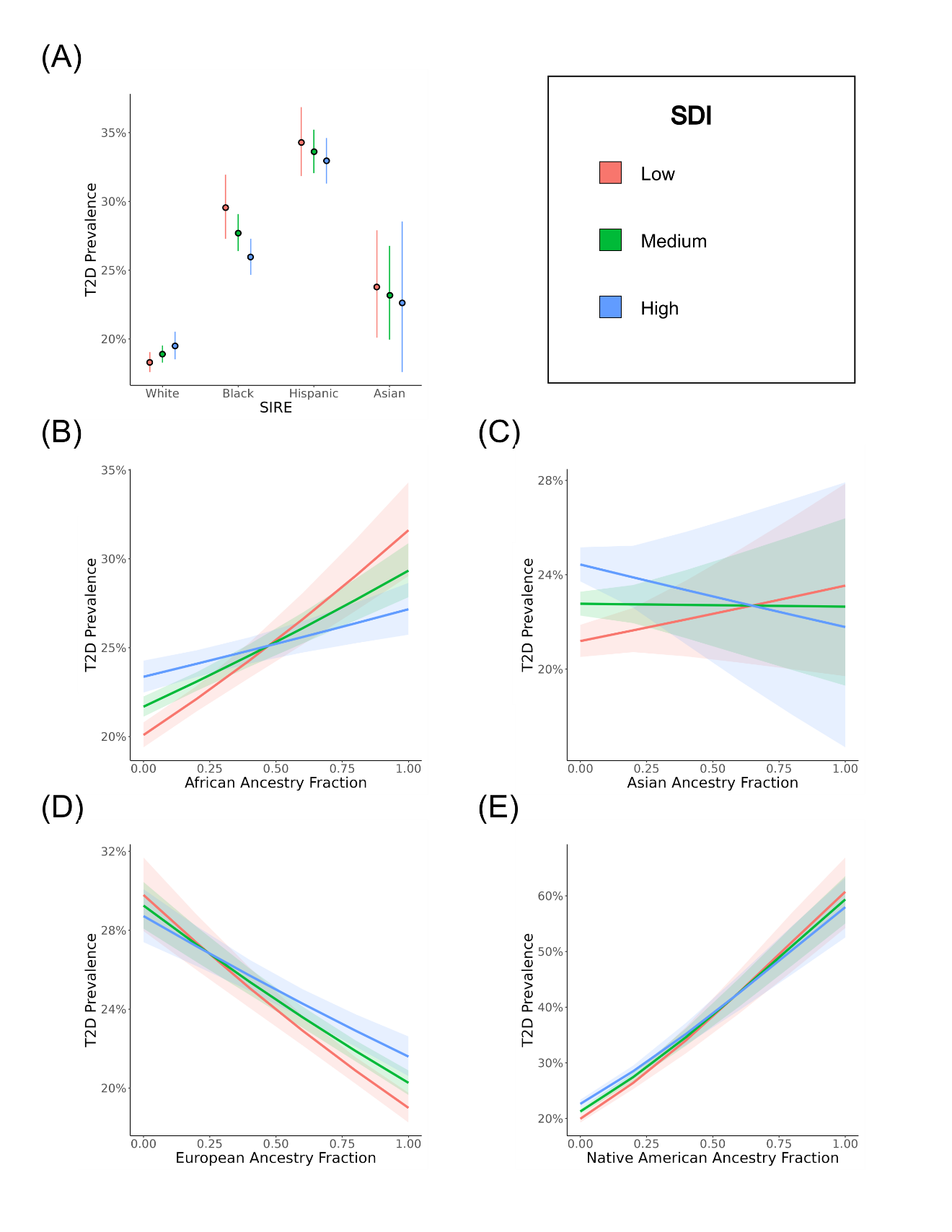


Fig. S3. **Male-stratified interaction effects between race/ethnicity, genetic ancestry, and socioeconomic deprivation (zSDI) on T2D prevalence.** T2D prevalence estimates, and 95% confidence intervals, are taken from multivariable logistic regression models that include interaction terms and are stratified by low (red), medium (green), and high (blue) zSDI. (A) Results for SIRE groups are based on the model T2D ~ SIRE*zSDI + age. (B-E) Results for genetic ancestry (GA) fractions are based on the model T2D ~ GA*zSDI + age.


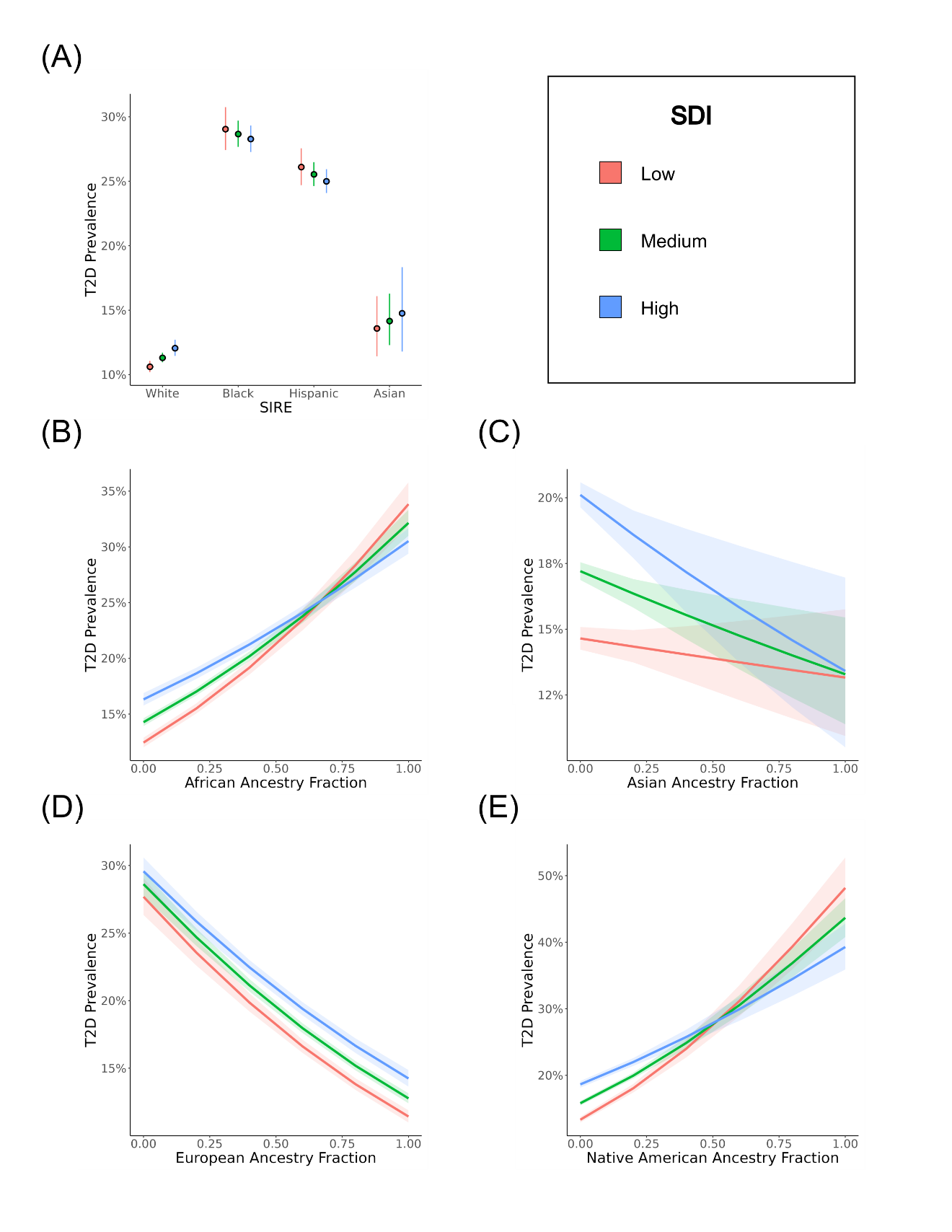


Fig. S4. **Female-stratified interaction effects between race/ethnicity, genetic ancestry, and socioeconomic deprivation (zSDI) on T2D prevalence.** T2D prevalence estimates, and 95% confidence intervals, are taken from multivariable logistic regression models that include interaction terms and are stratified by low (red), medium (green), and high (blue) zSDI. (A) Results for SIRE groups are based on the model T2D ~ SIRE*zSDI + age. (B-E) Results for genetic ancestry (GA) fractions are based on the model T2D ~ GA*zSDI + age.
